# Supplementary material for: Decoding the molecular, cellular, and functional heterogeneity of zebrafish intracardiac nervous system
Source: Nat Commun. 2024 Dec 4;15:10483. doi: 10.1038/s41467-024-54830-w (PMC11618350; doi:10.1038/s41467-024-54830-w)
Supplement: Supplementary file 1 — Supplementary Information [file 41467_2024_54830_MOESM1_ESM.pdf]

Supplementary Information:

Decoding the molecular, cellular, and functional heterogeneity of zebrafish intracardiac nervous system

Andrea Pedroni, Elanur Yilmaz, Lisa Del Vecchio, Prabesh Bhattarai, Inés Talaya Vidal, Yu-Wen E. Dai, Konstantinos Koutsogiannis, Caghan Kizil and Konstantinos Ampatzis

Supplementary Figures: 7
Supplementary Tables: 2

Supplementary Figures:

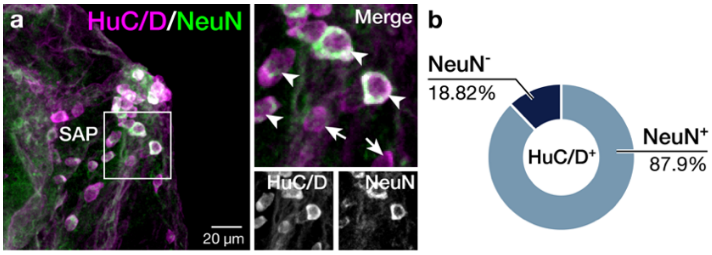

Supplementary Figure 1. Expression pattern of NeuN in the adult zebrafish SAP. a. Whole-mount photomicrographs showing the NeuN+ (green) and HuC/D+ (magenta) neuron somata in the SAP of the adult zebrafish heart. None of the neurons were found to be HuC/D-/NeuN+. Arrowheads indicate double-labeled neurons and arrows the HuC/D+/NeuN- **b**. The proportion of the HuC/D expressing neurons that also express NeuN. HuC/D, elav3+4; NeuN, neuronal protein (Fox-3/Rbfox3).

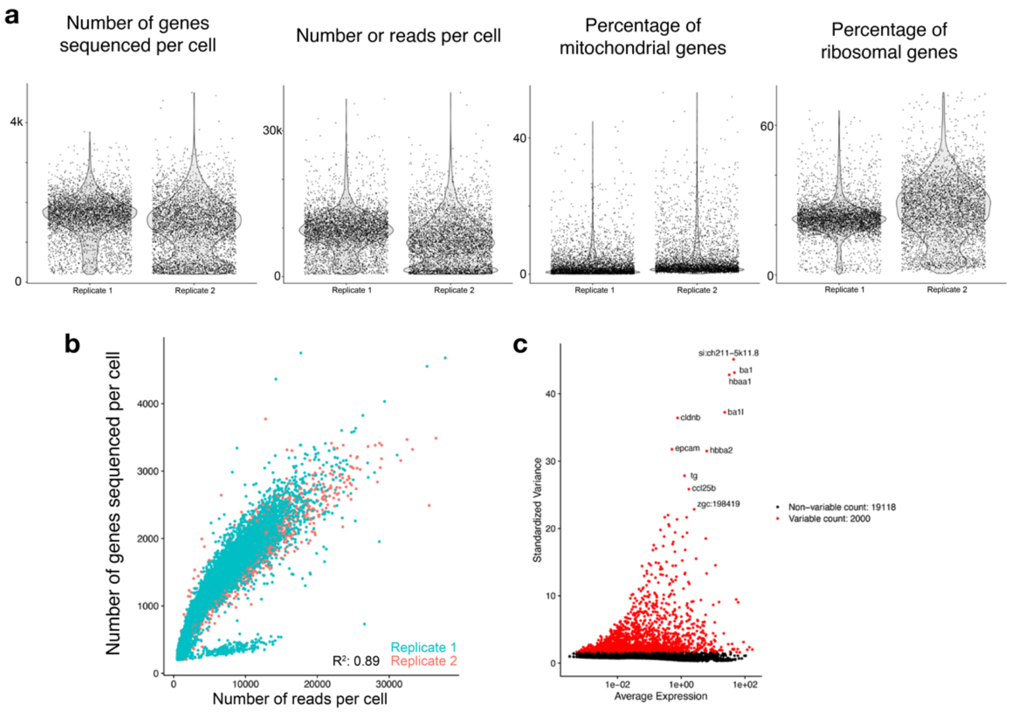

Supplementary Figure 2. **QC graphs for single-cell sequencing**. **a**. Violin plots for two single cell sequencing experiments (replicates). The number of genes sequenced per cell, number of reads per cell, and percentages of mitochondrial and ribosomal genes are shown. **b.** Distribution graph for the number of genes sequenced per cell and number of reads per cell. R^2: correlation coefficient. **c**. Volcano plot for the most variable genes in the entire dataset (red).

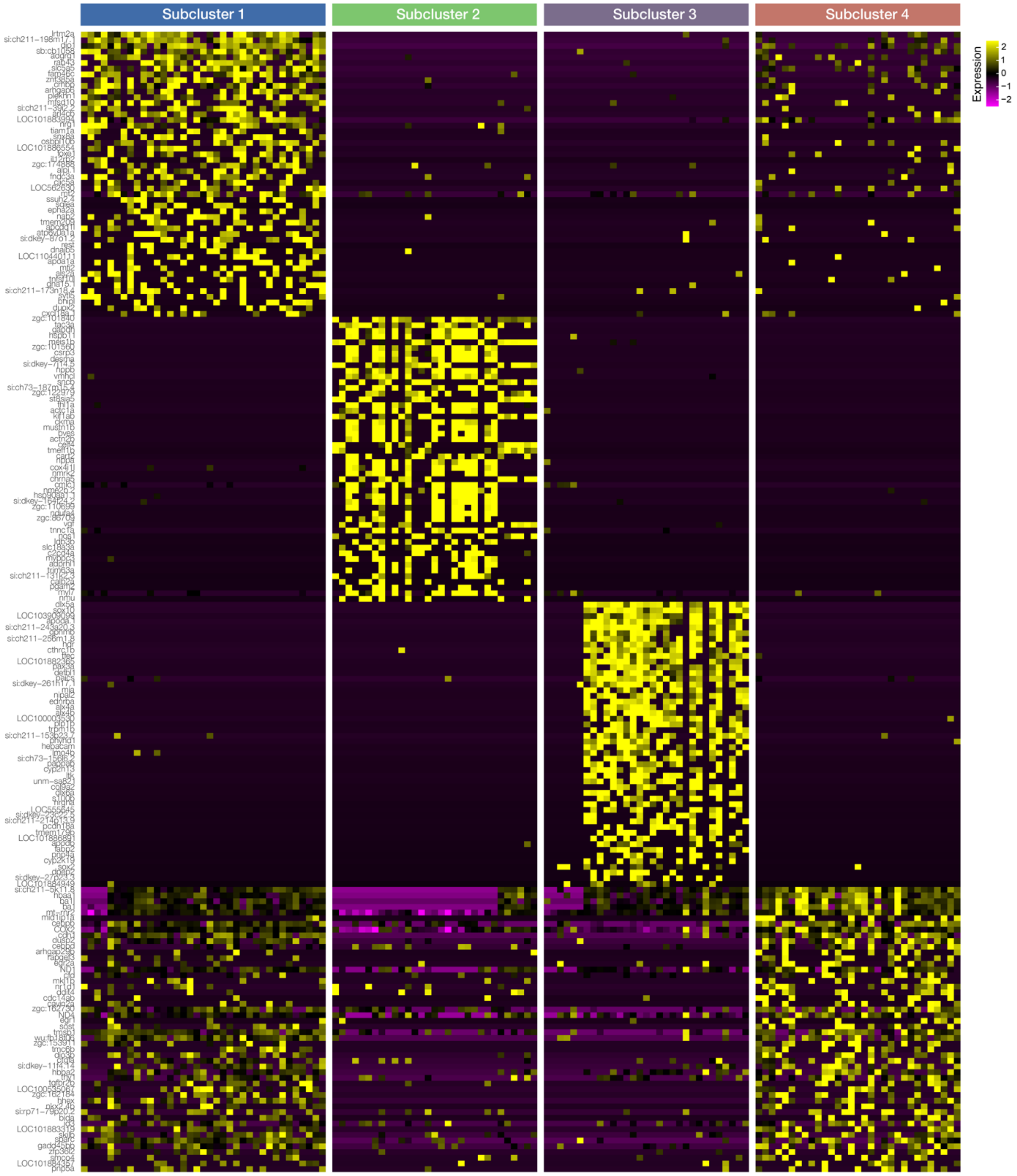

Supplementary Figure 3. Summary data for subclustering of the neural cell populations. Unbiased heat map of the top 50 genes of every subcluster. Every column indicates a cell and every row represents a particular gene. The different neuronal subclusters are indicated and color-coded.

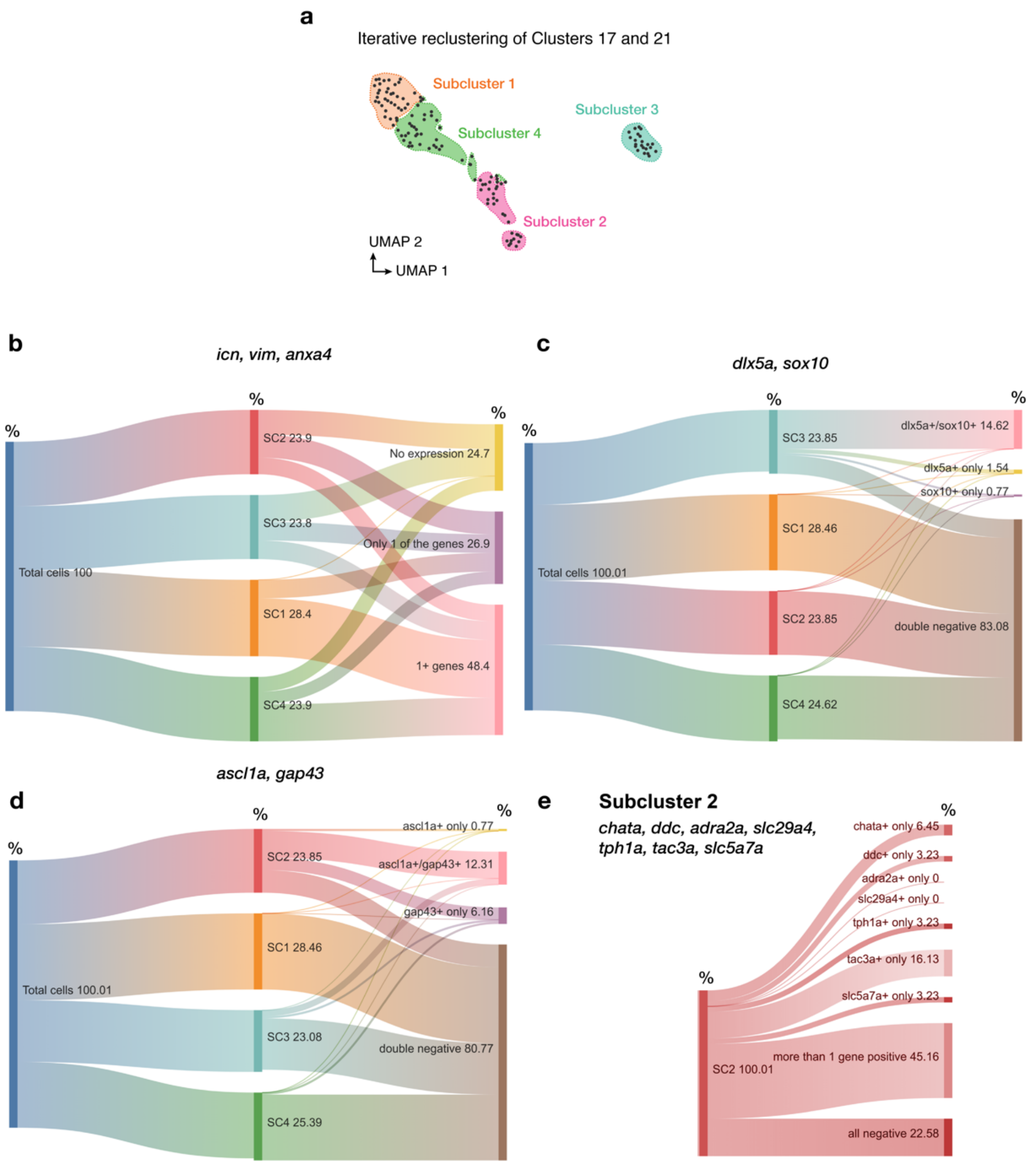

Supplementary Figure 4. Sankey diagrams for iteratively re-clustered nervous system-related cells. **a**. Reclustering of Clusters 17 and 21 resulted in 4 Subclusters (SCs). **b**. Cells analyzed regarding the expression of *icm, vim* and *anxa4*, ectodermal-epithelial markers. Most cells in Subclusters 1 and 4 express at least 1 of these neuroepithelial cells (100% of SC 1, 77.4% of SC 4). Subclusters 1 and 4 are the most representative of neuroepithelial signatures. **c.** Cells analyzed in regard to expression of *sox10* and *dlx5a*, Schwann cell markers. 71% of the cells in Subcluster 3 express at least 1 of these Schwann cell markers. Subcluster 3 represents Schwann cell signatures. **d.** Cells analyzed concerning the expression of *ascl1a* and *gap43*, neuronal markers. 64.5% of the cells in Subcluster 2 express at least 1 of these neuronal markers. Subcluster 2 represents neuronal cell signatures. **e**. As per expression of different neuronal subtype markers such as *chata, ddc, adra2a, slc29a4, tph1a, tac3a,* and *slc5a7a,* Subcluster 2 contains cholinergic, adrenergic, catecholaminergic, serotonergic, glutamatergic, and GABAergic neuronal types. 45.2% of Subcluster 2 expressed more than 1 of these genes, suggesting multiple neurotransmitter responsiveness.

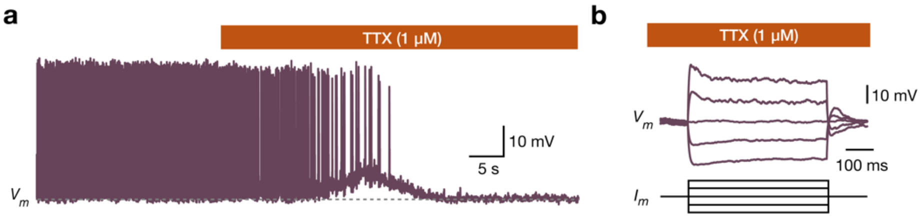

Supplementary Figure 5. SAP neurons release sodium-based action potentials. a-b. Voltage-clamp ramp recordings show that application of Na^+ channel blocker tetrodotoxin (TTX) eliminates the firing (action potentials) of SAP neurons (example from Bursting neuron, B), preserving the electrical properties (SAG and AHP rebound). TTX, tetrodotoxin.

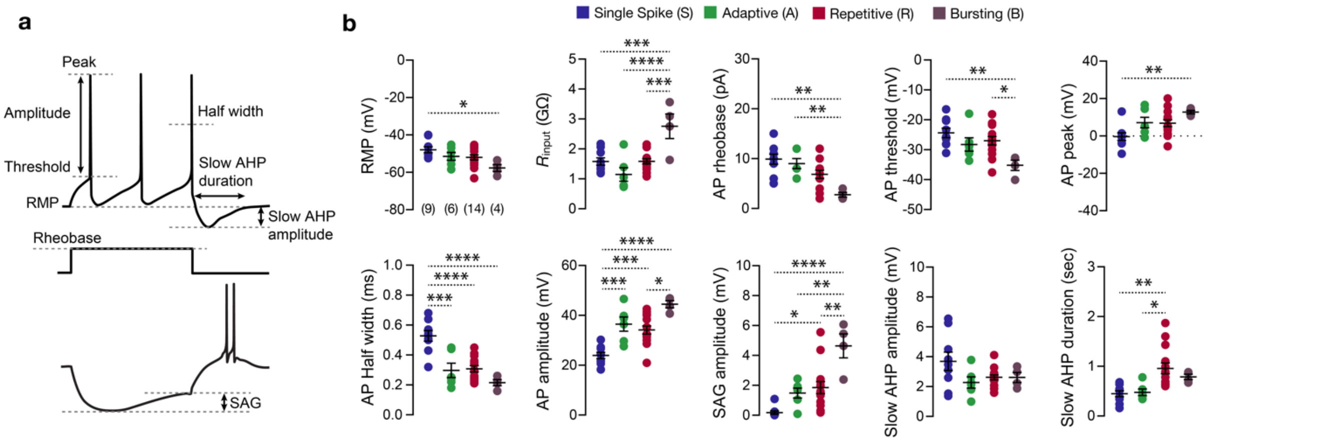

Supplementary Figure 6. Detained physiological features of the adult zebrafish SAP neurons. a. Schematic presentation of the measured electrical properties of the SAP neurons. **b.** Detailed analysis of the cellular and electrical properties of the SAP neurons in the adult zebrafish heart. AHP, after hyperpolarization potential; AP, action potential; *R*input, input resistance; RMP, resting membrane potential. Data are presented as mean±SEM. **P* < 0.05; ***P* < 0.01; ****P* < 0.001; *****P* < 0.0001. For detailed statistics, see Supplementary Table 1. Source data are provided as a Source Data file.

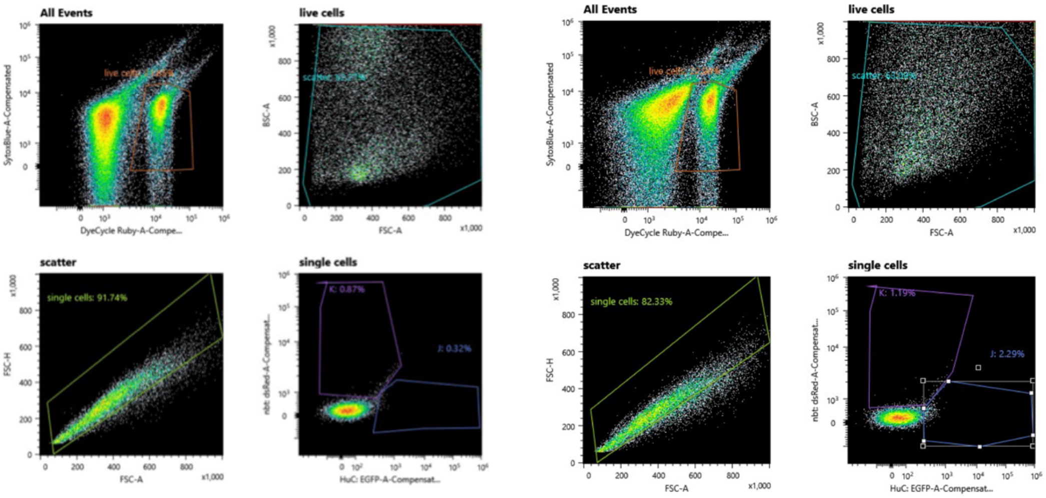

Gates and Statistics

Name	Events	%Parent	%Total
All Events	100,000	0.00%	100.00%
live cells	21,645	21.65%	21.65%
scatter	13,465	62.21%	13.47%
single cells	12,353	91.74%	12.35%
J	40	0.32%	0.04%
K	107	0.87%	0.11%

Gates and Statistics

Name	Events	%Parent	%Total
All Events	100,000	0.00%	100.00%
live cells	22,896	22.90%	22.90%
scatter	14,446	63.09%	14.45%
single cells	11,894	82.33%	11.89%
J	272	2.29%	0.27%
K	141	1.19%	0.14%

Supplementary Figure 7. Gating strategy for sorting GFP and DsRed-positive single cells. Plots demonstrate the gating strategy from WT-AB to separate GFP$^+$ and DsRed$^+$ cells (<1% of total cells) from negative cells (left panel) and sorting viable GFP$^+$ (approximately 2% of total cells in J-gating in single cells plot) and DsRed$^+$ (roughly 1% of total cells in K-gating in single cells plot) from transgenic *Tg(HuC:GFP)* and *Tg(nbt:dsRED)* lines (right panel). These lines were used to capture all possible neuronal populations in the heart. Rare cell populations sorted from J- and K-gatings were collected and pooled in a collection tube and processed for scRNA sequencing analysis.

Supplementary Table 1. Detailed statistics

Figure	Statistics	Result	Post-hoc Test	comparison	Significance	*P-value*
**Main figures**						
**1d**	Descriptive	SAP (*n* = 488 neurons): 39.79 ± 0.83<br>AVP (*n* = 75 neurons): 30.1 ± 1.61<br>VBAP (*n* = 33 neurons): 62.50 ± 2.06<br>BA wall (*n* = 18 neurons): 72.71 ± 2.26<br>Ventricle wall (*n* = 24 neurons): 77.65 ± 2.06				
**1e**	Descriptive	SAP neurons (*n* = 23 zebrafish): 81.04 ± 3.63				
**1f**	Linear regression	R^2 = 0.001<br>Sy.x = 16.28<br>Equation: Y= 2.696*X + 37.28<br>*n* = 239 neurons				
**3c**<br>Soma sizes	Unpaired *t*-test	t = 5.13, df = 206 (Two-tailed)		ChAT$^+$<br>ChAT$^-$	****	$P < 0.0001$
	Descriptive	ChAT$^+$ (*n* = 119 neurons): 45.47 ± 1.56<br>ChAT$^-$ (*n* = 89 neurons): 35.15 ± 1.00				
**3i**<br>Proportion	Descriptive	Cholinergic (*n* = 16 zebrafish): 80.96 ± 0.99<br>Glutamatergic (*n* = 9 zebrafish): 8.26 ± 0.44<br>GABAergic (*n* = 6 zebrafish): 6.41 ± 0.34<br>Serotonergic (*n* = 6 zebrafish): 5.05 ± 0.39<br>Catecholaminergic (*n* = 7 zebrafish): 4.58 ± 0.29				
**3i**<br>Soma size	One-way ANOVA	$F_{(4, 203)} = 7.259$<br>$P < 0.0001$	Tukey's test	Cholinergic<br>Glutamatergic	**	$P_{adj} = 0.0035$
				Cholinergic<br>GABAergic	***	$P_{adj} = 0.0002$
				Cholinergic<br>Serotonergic	ns	$P_{adj} = 0.1205$
				Cholinergic<br>Catecholaminergic	ns	$P_{adj} = 0.5108$
				Glutamatergic<br>GABAergic	ns	$P_{adj} = 0.7493$
				Glutamatergic<br>Serotonergic	ns	$P_{adj} > 0.9999$
				Glutamatergic<br>Catecholaminergic	ns	$P_{adj} = 0.9554$
				GABAergic<br>Serotonergic	ns	$P_{adj} = 0.9258$
				GABAergic<br>Catecholaminergic	ns	$P_{adj} = 0.5114$
				Serotonergic<br>Catecholaminergic	ns	$P_{adj} = 0.9652$
	Descriptive	Cholinergic (*n* = 119 neurons): 45.47 ± 1.56<br>Glutamatergic (*n* = 39 neurons): 35.9 ± 1.17<br>GABAergic (*n* = 23 neurons): 31.36 ± 2.4<br>Serotonergic (*n* = 13 neurons): 35.42 ± 1.84<br>Catecholaminergic (*n* = 14 neurons): 39.05 ± 3.19				
**4c**<br>Firing %	Descriptive	Single Spike (S): *n* = 9 neurons<br>Adaptive (A): *n* = 6 neurons<br>Repetitive (R): *n* = 14 neurons<br>Bursting (B): *n* = 4 neurons				
**4e**<br>Spontaneous firing %	Descriptive	Single Spike (S): *n* = 0 out of 9 neurons<br>Adaptive (A): *n* = 2 out of 6 neurons<br>Repetitive (R): *n* = 9 out of 14 neurons<br>Bursting (B): *n* = 4 out of 4 neurons				
**4e**<br>Firing frequency	One-way ANOVA	$F_{(3, 29)} = 110.3$<br>$P < 0.0001$	Tukey's test	Single Spike (S)<br>Adaptive (A)	ns	$P_{adj} = 0.9593$
				Single Spike (S)<br>Repetitive (R)	*	$P_{adj} = 0.0127$
				Single Spike (S)<br>Bursting (B)	****	$P_{adj} < 0.0001$
				Adaptive (A)<br>Repetitive (R)	ns	$P_{adj} = 0.1078$

				Adaptive (A)<br>Bursting (B)	****	$P_{adj} < 0.0001$
				Repetitive (R)<br>Bursting (B)	****	$P_{adj} < 0.0001$
	Descriptive	Single Spike (S) (*n* = 9 neurons): 0.0 ± 0.0<br>Adaptive (A) (*n* = 6 neurons): 0.12 ± 0.1<br>Repetitive (R) (*n* = 14 neurons): 0.64 ± 0.15<br>Bursting (B) (*n* = 4 neurons): 4.7 ± 0.32				
**4f**<br>EPSC frequency	One-way ANOVA	$F_{(3, 15)} = 0.5344$			ns	$P = 0.6657$
	Descriptive	Single Spike (S) (*n* = 5 neurons): 1.32 ± 0.25<br>Adaptive (A) (*n* = 5 neurons): 0.98 ± 0.12<br>Repetitive (R) (*n* = 6 neurons): 1.28 ± 0.2<br>Bursting (B) (*n* = 3 neurons): 1.13 ± 0.26				
**4f**<br>Mean EPSC amplitude	One-way ANOVA	$F_{(3, 15)} = 1.138$			ns	$P = 0.3655$
	Descriptive	Single Spike (S) (*n* = 5 neurons): 21.11 ± 0.68<br>Adaptive (A) (*n* = 5 neurons): 21.83 ± 1.72<br>Repetitive (R) (*n* = 6 neurons): 20.23 ± 1.07<br>Bursting (B) (*n* = 3 neurons): 24.26 ± 2.75				
**4f**<br>EPSC amplitude	One-way ANOVA	$F_{(3, 536)} = 1.406$			ns	$P = 0.2402$
	Descriptive	Single Spike (S) (*n* = 143 EPSCs): 21.08 ± 1.41<br>Adaptive (A) (*n* = 133 EPSCs): 21.97 ± 1.58<br>Repetitive (R) (*n* = 167 EPSCs): 20.26 ± 1.18<br>Bursting (B) (*n* = 97 EPSCs): 24.74 ± 2.14				
**5c**	Unpaired *t*-test	t = 0.687, df = 28 (Two-tailed)		*Ex-vivo*<br>Cardiomyocyte	ns	$P = 0.4974$
	Descriptive	*Ex-vivo* freq. (*n* = 16 hearts): 2.88 ± 0.11<br>Cardiomyocyte freq. (*n* = 14 cells): 2.75 ± 0.14				
**5d**	Paired *t*-test	t = 15.97, df = 5 (Two-tailed)		Before sucrose<br>After sucrose	****	$P < 0.0001$
	Descriptive	Before sucrose (*n* = 6 neurons): 1.07 ± 0.06<br>After sucrose (*n* = 6 neurons): 31.83 ± 1.88				
**5e**	Paired *t*-test	t = 19.04, df = 6 (Two-tailed)		Before sucrose<br>After sucrose	****	$P < 0.0001$
	Descriptive	Before sucrose (*n* = 7 cardiomyocytes): 2.94 ± 0.09<br>After sucrose (*n* = 7 cardiomyocytes): 0.31 ± 0.13				
**5g**	Paired *t*-test	t = 10.79, df = 4 (Two-tailed)		Before sucrose<br>After sucrose	***	$P = 0.0004$
	Descriptive	Before sucrose (*n* = 5 hearts): 3.16 ± 0.1<br>After sucrose (*n* = 5 hearts): 2.87 ± 0.09				
**5h**	Unpaired *t*-test	t = 1.971, df = 21 (Two-tailed)		Control<br>Reduced	ns	$P = 0.0620$
	Descriptive	Control (*n* = 11 hearts): 3.11 ± 0.1<br>Reduced (*n* = 12 hearts): 2.73 ± 0.15				
**5i**	Paired *t*-test	t = 2.564, df = 5 (Two-tailed)		Before sucrose<br>After sucrose	ns	$P = 0.0504$
	Descriptive	Before sucrose (*n* = 6 hearts): 2.88 ± 0.09<br>After sucrose (*n* = 6 hearts): 2.7 ± 0.03				
**Supplementary figures**						
**Supplementary 6**<br>RMP	One-way ANOVA	$F_{(3, 29)} = 3.836$<br>$P = 0.0199$	Tukey's test	Single Spike (S)<br>Adaptive (A)	ns	$P_{adj} = 0.5243$
				Single Spike (S)<br>Repetitive (R)	ns	$P_{adj} = 0.2298$
				Single Spike (S)<br>Bursting (B)	*	$P_{adj} = 0.0116$
				Adaptive (A)<br>Repetitive (R)	ns	$P_{adj} = 0.9960$
				Adaptive (A)<br>Bursting (B)	ns	$P_{adj} = 0.2143$
				Repetitive (R)<br>Bursting (B)	ns	$P_{adj} = 0.1847$
	Descriptive	Single Spike (S) (*n* = 9 neurons): -47.96 ± 1.59<br>Adaptive (A) (*n* = 6 neurons): -51.47 ± 2.1				

		Repetitive (R) (*n* = 14 neurons): -52.0 ± 1.33<br>Bursting (B) (*n* = 4 neurons): -57.7 ± 1.82				
**Supplementary 6**<br>Rinput	One-way ANOVA	$F_{(3, 29)} = 10.54$<br>$P < 0.0001$	Tukey's test	Single Spike (S)<br>Adaptive (A)	ns	$P_{adj} = 0.2857$
				Single Spike (S)<br>Repetitive (R)	ns	$P_{adj} > 0.9999$
				Single Spike (S)<br>Bursting (B)	***	$P_{adj} = 0.0009$
				Adaptive (A)<br>Repetitive (R)	ns	$P_{adj} = 0.2138$
				Adaptive (A)<br>Bursting (B)	****	$P_{adj} < 0.0001$
				Repetitive (R)<br>Bursting (B)	***	$P_{adj} = 0.0005$
	Descriptive	Single Spike (S) (*n* = 9 neurons): 1.58 ± 0.12<br>Adaptive (A) (*n* = 6 neurons): 1.14 ± 0.22<br>Repetitive (R) (*n* = 14 neurons): 1.58 ± 0.08<br>Bursting (B) (*n* = 4 neurons): 2.75 ± 0.41				
**Supplementary 6**<br>Rheobase	One-way ANOVA	$F_{(3, 29)} = 6.773$<br>$P = 0.0013$	Tukey's test	Single Spike (S)<br>Adaptive (A)	ns	$P_{adj} = 0.9313$
				Single Spike (S)<br>Repetitive (R)	ns	$P_{adj} = 0.0772$
				Single Spike (S)<br>Bursting (B)	**	$P_{adj} = 0.0012$
				Adaptive (A)<br>Repetitive (R)	ns	$P_{adj} = 0.4155$
				Adaptive (A)<br>Bursting (B)	**	$P_{adj} = 0.0091$
				Repetitive (R)<br>Bursting (B)	ns	$P_{adj} = 0.0690$
	Descriptive	Single Spike (S) (*n* = 9 neurons): 9.88 ± 1.0<br>Adaptive (A) (*n* = 6 neurons): 9.0 ± 1.0<br>Repetitive (R) (*n* = 14 neurons): 6.85 ± 0.82<br>Bursting (B) (*n* = 4 neurons): 2.75 ± 0.47				
**Supplementary 6**<br>AP threshold	One-way ANOVA	$F_{(3, 29)} = 4.411$<br>$P = 0.0113$	Tukey's test	Single Spike (S)<br>Adaptive (A)	ns	$P_{adj} = 0.4702$
				Single Spike (S)<br>Repetitive (R)	ns	$P_{adj} = 0.6210$
				Single Spike (S)<br>Bursting (B)	**	$P_{adj} = 0.0062$
				Adaptive (A)<br>Repetitive (R)	ns	$P_{adj} = 0.9542$
				Adaptive (A)<br>Bursting (B)	ns	$P_{adj} = 0.1615$
				Repetitive (R)<br>Bursting (B)	*	$P_{adj} = 0.0342$
	Descriptive	Single Spike (S) (*n* = 9 neurons): -24.38 ± 1.57<br>Adaptive (A) (*n* = 6 neurons): -28.26 ± 2.23<br>Repetitive (R) (*n* = 14 neurons): -26.99 ± 1.41<br>Bursting (B) (*n* = 4 neurons): -35.22 ± 1.72				
**Supplementary 6**<br>AP Half width duration	One-way ANOVA	$F_{(3, 29)} = 15.75$<br>$P < 0.0001$	Tukey's test	Single Spike (S)<br>Adaptive (A)	***	$P_{adj} = 0.0003$
				Single Spike (S)<br>Repetitive (R)	****	$P_{adj} < 0.0001$
				Single Spike (S)<br>Bursting (B)	****	$P_{adj} < 0.0001$
				Adaptive (A)<br>Repetitive (R)	ns	$P_{adj} = 0.9959$
				Adaptive (A)<br>Bursting (B)	ns	$P_{adj} = 0.5206$
				Repetitive (R)<br>Bursting (B)	ns	$P_{adj} = 0.3096$
	Descriptive	Single Spike (S) (*n* = 9 neurons): 0.52 ± 0.03<br>Adaptive (A) (*n* = 6 neurons): 0.29 ± 0.04<br>Repetitive (R) (*n* = 14 neurons): 0.3 ± 0.02				

		Bursting (B) (n = 4 neurons): 0.21 ± 0.02				
**Supplementary 6**<br>AP amplitude	One-way ANOVA	$F_{(3, 29)} = 15.54$<br>$P < 0.0001$	Tukey's test	Single Spike (S)<br>Adaptive (A)	***	$P_{adj} = 0.0007$
				Single Spike (S)<br>Repetitive (R)	***	$P_{adj} = 0.0007$
				Single Spike (S)<br>Bursting (B)	****	$P_{adj} < 0.0001$
				Adaptive (A)<br>Repetitive (R)	ns	$P_{adj} = 0.8071$
				Adaptive (A)<br>Bursting (B)	ns	$P_{adj} = 0.1243$
				Repetitive (R)<br>Bursting (B)	*	$P_{adj} = 0.0107$
	Descriptive	Single Spike (S) (n = 9 neurons): 23.91 ± 1.23<br>Adaptive (A) (n = 6 neurons): 36.49 ± 2.85<br>Repetitive (R) (n = 14 neurons): 34.13 ± 1.6<br>Bursting (B) (n = 4 neurons): 44.47 ± 1.43				
**Supplementary 6**<br>SAG amplitude	One-way ANOVA	$F_{(3, 29)} = 13.06$<br>$P < 0.0001$	Tukey's test	Single Spike (S)<br>Adaptive (A)	ns	$P_{adj} = 0.1853$
				Single Spike (S)<br>Repetitive (R)	*	$P_{adj} = 0.0137$
				Single Spike (S)<br>Bursting (B)	****	$P_{adj} < 0.0001$
				Adaptive (A)<br>Repetitive (R)	ns	$P_{adj} = 0.9220$
				Adaptive (A)<br>Bursting (B)	**	$P_{adj} = 0.0017$
				Repetitive (R)<br>Bursting (B)	**	$P_{adj} = 0.0016$
	Descriptive	Single Spike (S) (n = 9 neurons): 0.17 ± 0.11<br>Adaptive (A) (n = 6 neurons): 1.48 ± 0.33<br>Repetitive (R) (n = 14 neurons): 1.85 ± 0.4<br>Bursting (B) (n = 4 neurons): 4.63 ± 0.8				
**Supplementary 6**<br>Slow AHP amplitude	One-way ANOVA	$F_{(3, 29)} = 2.398$			ns	$P = 0.0883$
	Descriptive	Single Spike (S) (n = 9 neurons): 3.69 ± 0.61<br>Adaptive (A) (n = 6 neurons): 2.27 ± 0.37<br>Repetitive (R) (n = 14 neurons): 2.61 ± 0.16<br>Bursting (B) (n = 4 neurons): 2.6 ± 0.33				
**Supplementary 6**<br>Slow AHP duration	One-way ANOVA	$F_{(3, 29)} = 6.90$<br>$P = 0.0012$	Tukey's test	Single Spike (S)<br>Adaptive (A)	ns	$P_{adj} = 0.9976$
				Single Spike (S)<br>Repetitive (R)	**	$P_{adj} = 0.002$
				Single Spike (S)<br>Bursting (B)	ns	$P_{adj} = 0.2516$
				Adaptive (A)<br>Repetitive (R)	*	$P_{adj} = 0.0124$
				Adaptive (A)<br>Bursting (B)	ns	$P_{adj} = 0.3872$
				Repetitive (R)<br>Bursting (B)	ns	$P_{adj} = 0.7408$
	Descriptive	Single Spike (S) (n = 9 neurons): 0.44 ± 0.05<br>Adaptive (A) (n = 6 neurons): 0.47 ± 0.06<br>Repetitive (R) (n = 14 neurons): 0.95 ± 0.1<br>Bursting (B) (n = 4 neurons): 0.78 ± 0.05				

Supplementary Table 2. Antibodies and Streptavidins Used[1]

Antigen	Host	Source	Code	Dilution
Primary				
α2B-ARs	Mouse	Santa Cruz	sc-390430	1:100
ChAT	Goat	Millipore	AB144P; RRID: AB_2079751	1:200
GABA	Rabbit	Sigma	A2052; RRID: AB_477652	1:2000
Gad1b	Rabbit	GeneTex	GTX124355; RRID: AB_11167690	1:50-1:100
GFP	Chicken	Abcam	AB13970; RRID: AB_300798	1:500
HuC/D	Mouse	Molecular Probes	A-21271; RRID: AB_221448	1:500
HuC/D	Rabbit	GeneTex	GTX128365; RRID: AB_2885764	1:400
NeuN	Rabbit	Cell Signaling	#24307; RRID: AB_2651140	1:400
TH	Mouse	Millipore	MAB318; RRID: AB_2201528	1:800
Zn-12	Mouse	DSHB	RRID: AB_531908	1:100
5-HT	Rabbit	Sigma	S5545; RRID: AB_477522	1:2000
Secondary				
Goat IgG-488	Donkey	ThermoFisher	A-11055; RRID: AB_2534102	1:500
Mouse IgG-647	Donkey	ThermoFisher	A-31571; RRID: AB_162542	1:500
Mouse IgG-568	Donkey	ThermoFisher	A-10037; RRID: AB_2534013	1:500
Mouse IgG-488	Donkey	ThermoFisher	A-21202; RRID: AB_141607	1:500
Rabbit IgG-488	Donkey	ThermoFisher	A-21206; RRID: AB_2535792	1:500
Rabbit IgG-647	Donkey	ThermoFisher	A-31573; RRID: AB_2536183	1:500
Rabbit IgG-568	Donkey	ThermoFisher	A-10042; RRID: AB_2534017	1:500
Mouse IgG-Biotinylated	Horse	VectorLaboratories	BA-2000; RRID: AB_2313581	1:200
Rabbit IgG-Biotinylated	Horse	VectorLaboratories	BA-1100; RRID: AB_2336201	1:200
Streptavidin				
Alexa Fluor 488		ThermoFisher	S32354; RRID: AB_2315383	1:500
Alexa Fluor 555		ThermoFisher	S32355; RRID: AB_2571525	1:500
Alexa Fluor 647		ThermoFisher	S32357; RRID: AB_2336066	1:500

[1]5-HT, 5-hydroxytryptamine (Serotonin); α2B-ARs, alpha2B-adrenergic receptors; ChAT, choline-acetyltransferase; GABA, γ-aminobutyric acid; Gad1, Glutamate Decarboxylase 1; GFP, green fluorescent protein; HuC/D, Elav3+4; NeuN, Neuronal protein Fox-3/RBFOX3; TH, tyrosine hydroxylase; Zn-12, neuronal cell surface marker (HNK-1 carbohydrate epitope).
