# Supplementary material for: Decoding the molecular, cellular, and functional heterogeneity of zebrafish intracardiac nervous system
Source: Nat Commun. 2024 Dec 4;15:10483. doi: 10.1038/s41467-024-54830-w (PMC11618350; doi:10.1038/s41467-024-54830-w)
Supplement: Supplementary file 2 — Description of Additional Supplementary Files [file 41467_2024_54830_MOESM2_ESM.pdf]

Description of Additional Supplementary Files

Supplementary Dataset 1

Flow cytometry sorting gating properties, histograms, and cell percentages of fluorescently labeled cells.

Supplementary Dataset 2

The genes expressed in the Schwann cell cluster prioritized according to the fold change. Gene expression levels in Schwann cells were identified using the Seurat pipeline. Following a data normalization to account for variations in sequencing depth across cells, Schwann cells were identified by clustering based on shared nearest neighbor (SNN) graph construction and visualization through Uniform Manifold Approximation and Projection (UMAP). Expressed genes and their representation fold changes were identified using Seurat and DESeq2, applying a negative binomial model to the normalized counts. The Wald test assessed statistical significance, and P values were adjusted using the Benjamini-Hochberg method to control the false discovery rate (FDR < 0.05).

Supplementary Dataset 3

Differentially expressed genes in neurons compared to other cell types. Differential expression analysis to identify neuron-specific genes using Seurat. Normalized single-cell RNA sequencing data were analyzed, and differential expression was assessed using DESeq2. The Wald test was applied to identify significant changes in gene expression between neurons and other cell types, with p-values adjusted using the Benjamini-Hochberg method to control for the false discovery rate (FDR < 0.05). Genes are ranked by log fold change, highlighting neuron-enriched transcripts in the dataset.
