# Supplementary material for: Decoding the molecular, cellular, and functional heterogeneity of zebrafish intracardiac nervous system
Source: Nat Commun. 2024 Dec 4;15:10483. doi: 10.1038/s41467-024-54830-w (PMC11618350; doi:10.1038/s41467-024-54830-w)
Supplement: Supplementary file 4 — Supplementary Dataset 1 [file 41467_2024_54830_MOESM4_ESM.pdf]

Wild type - Non-fluorescent cell sorting for gating adjustment

Experiment Information

Name	Experiment 9/1/2023 3:04:38 PM
Date	9/1/2023 3:04:38 PM
Model Type	LE-MA900FP
Serial Number	716004
Investigator	
Operator	
Memo	

Sample Group Information

Name	Sample Group - 1
Species	
Cell Type	
Memo	

Measurement Settings

Parameter Settings

	Marker	Fluorochrome	Acquisition Select Area	Height	Width
FSC	---	---	✓	✓	✓
BSC	---	---	✓		
FL1	HuC	EGFP	✓		
FL2	nbt	dsRed	✓		
FL3		PE-Texas Red			
FL4		PerCP-Cy5.5			
FL5		PE-Cy7			
FL6		SytoxBlue	✓		
FL7		Brilliant Violet 510			
FL8		Brilliant Violet 570			
FL9		Brilliant Violet 605			
FL10		DyeCycle Ruby	✓		
FL11		Alexa Fluor 700			
FL12		APC-Alexa Fluor 750			

Compensation Settings

Spillover Matrix (%)

Detector

Fluorochrome	HuC: EGFP	nbt: dsRed	SytoxBlue	DyeCycle Ruby
HuC: EGFP	100.00	5.14	0.00	0.00
nbt: dsRed	-0.44	100.00	0.00	0.00
SytoxBlue	0.00	0.00	100.00	0.00
DyeCycle Ruby	0.00	0.00	0.00	100.00

Negative Value

Detector

Area	0	0	0	0
Height	0	0	0	0

Tube Information

Name	WT
Sample ID1	
Sample ID2	
Sample ID3	
Sample ID4	

Basic Information

Description	
Model Type	LE-MA900FP
Serial Number	716004
Chip Type	Unknown
Chip ID	
Nozzle Size	Unknown

Sorting Setting

Sorting Method 5 mL Tubes

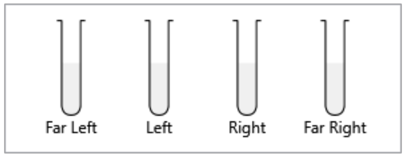

Collection Tube	Sort Gate	Sort Mode	Cell Size	Stop Count

Worksheet

All Events

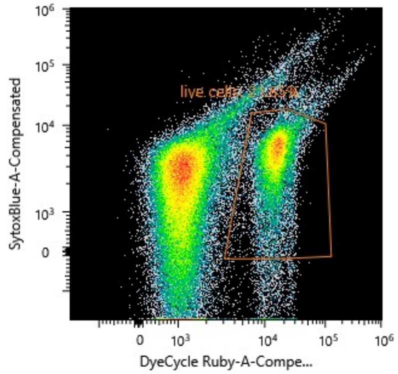

live cells

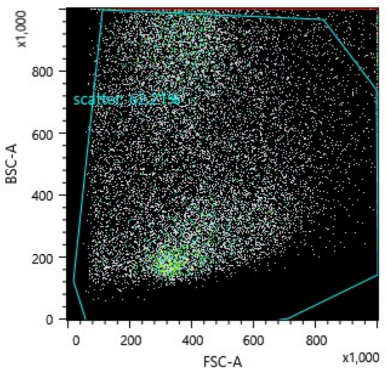

scatter

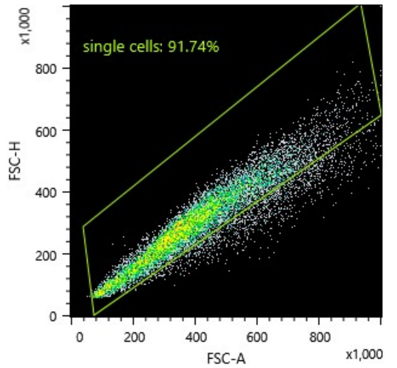

single cells

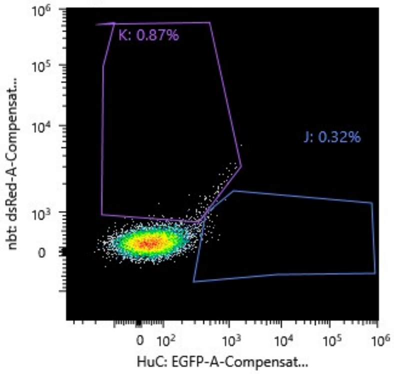

Gates and Statistics

Name	Events	%Parent	%Total
All Events	100,000	0.00%	100.00%
live cells	21,645	21.65%	21.65%
scatter	13,465	62.21%	13.47%
single cells	12,353	91.74%	12.35%
J	40	0.32%	0.04%
K	107	0.87%	0.11%

Gates and Statistics

Name	Events	%Parent	%Total
All Events	100,000	0.00%	100.00%
live cells	21,645	21.65%	21.65%
scatter	13,465	62.21%	13.47%
single cells	12,353	91.74%	12.35%
J	40	0.32%	0.04%
K	107	0.87%	0.11%

Fluorescent cell sorting from transgenic animals

Experiment Information

Name	Experiment 9/1/2023 3:04:38 PM
Date	9/1/2023 3:04:38 PM
Model Type	LE-MA900FP
Serial Number	716004
Investigator	
Operator	
Memo	

Sample Group Information

Name	Sample Group - 1
Species	
Cell Type	
Memo	

Measurement Settings

Parameter Settings

	Marker	Fluorochrome	Acquisition Select Area	Height	Width
FSC	---	---	✓	✓	✓
BSC	---	---	✓		
FL1	HuC	EGFP	✓		
FL2	nbt	dsRed	✓		
FL3		PE-Texas Red			
FL4		PerCP-Cy5.5			
FL5		PE-Cy7			
FL6		SytoxBlue	✓		
FL7		Brilliant Violet 510			
FL8		Brilliant Violet 570			
FL9		Brilliant Violet 605			
FL10		DyeCycle Ruby	✓		
FL11		Alexa Fluor 700			
FL12		APC-Alexa Fluor 750			

Compensation Settings

Spillover Matrix (%)

Detector

Fluorochrome	HuC: EGFP	nbt: dsRed	SytoxBlue	DyeCycle Ruby
HuC: EGFP	100.00	5.14	0.00	0.00
nbt: dsRed	-0.44	100.00	0.00	0.00
SytoxBlue	0.00	0.00	100.00	0.00
DyeCycle Ruby	0.00	0.00	0.00	100.00

Negative Value

Detector

Area	0	0	0	0
Height	0	0	0	0

Tube Information

Name	Heart
Sample ID1	
Sample ID2	
Sample ID3	
Sample ID4	

Basic Information

Description	
Model Type	LE-MA900FP
Serial Number	716004
Chip Type	Sorting Chip
Chip ID	00404860-0000-7060
Nozzle Size	100 µm

Sample Stop Condition

None

Recording Setting

Type	Event Count
Value	10,000,000

Instrument Setting

Laser

405nm	On
488nm	On
561nm	On
638nm	On

Threshold

Channel:	FSC
Value:	5.00%

Sensor Gain

FSC	13	BSC	37.5%
FL1	37.0%	FL2	43.0%
FL3	40.0%	FL4	40.0%
FL5	40.0%	FL6	53.5%
FL7	40.0%	FL8	40.0%
FL9	40.0%	FL10	48.5%
FL11	40.0%	FL12	40.0%

Sample Pressure 8

Auto Parameters

Droplet Clock	29,400	Hz
Droplet Drive	8.26	
Sort Delay	24	
Sort Phase	180	deg
Charge	80.0	%
Deflection FarLeft	-1,044	
Deflection Left	-489	
Deflection Right	424	
Deflection FarRight	1,014	
Enabled Control Breakoff	✔	

Sorting Setting

Sorting Method 5 mL Tubes

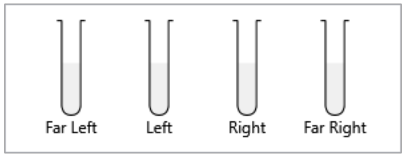

Collection Tube	Sort Gate	Sort Mode	Cell Size	Stop Count
Left	J	Purity	Regular Cell	0
Right	K	Purity	Regular Cell	0

Worksheet

All Events

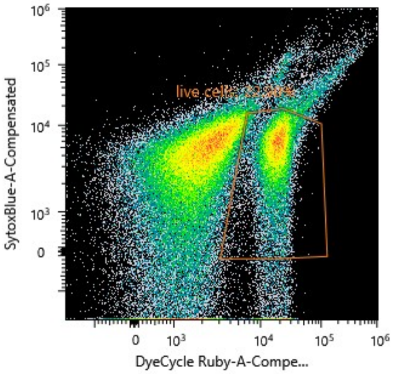

live cells

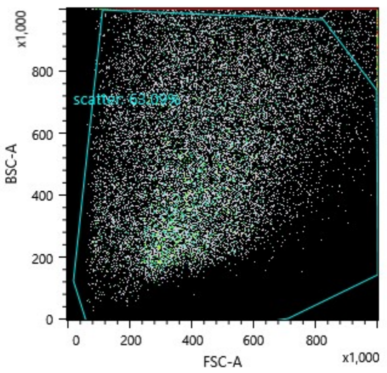

scatter

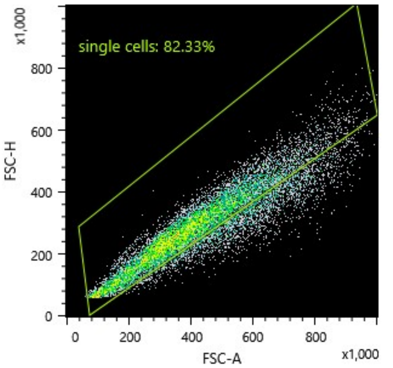

single cells

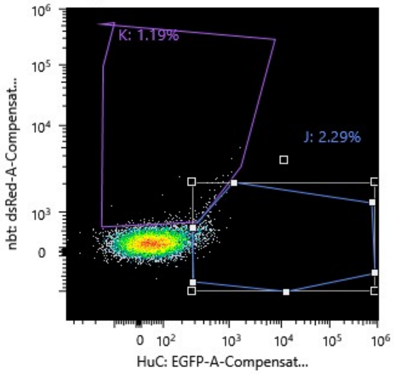

Gates and Statistics

Displaying 100,000 / 6,999,620 events

Name	Events	%Parent	%Total
All Events	100,000	0.00%	100.00%
live cells	22,896	22.90%	22.90%
scatter	14,446	63.09%	14.45%
single cells	11,894	82.33%	11.89%
J	272	2.29%	0.27%
K	141	1.19%	0.14%

Gates and Statistics

Name	Events	%Parent	%Total
All Events	100,000	0.00%	100.00%
live cells	22,896	22.90%	22.90%
scatter	14,446	63.09%	14.45%
single cells	11,894	82.33%	11.89%
J	272	2.29%	0.27%
K	141	1.19%	0.14%

Recording Result

Total Record Count:	6,999,620
Start Time:	9/1/2023 3:23:17 PM
End Time:	9/1/2023 3:39:28 PM

Sorting Result

Sorting Method 5 mL Tubes

Start Time 9/1/2023 3:23:17 PM

End Time 9/1/2023 3:39:28 PM

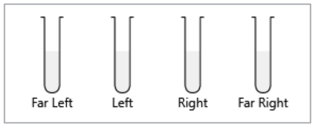

Collection Tube	Sort Gate	Sort Mode	Elapsed Time	Total Event	Target Ratio	Sorted Count	Sort Rate	Sort Efficiency	Aborted Count	Abort Rate
Left	J	Purity	00:16:10	7,308,825	0.27%	11,033	11.37eps	56.52%	8,488	8.74eps
Right	K	Purity	00:16:10	7,308,825	0.14%	6,156	6.34eps	60.56%	4,009	4.13eps
