# Supplementary material for: Decoding the molecular, cellular, and functional heterogeneity of zebrafish intracardiac nervous system
Source: Nat Commun. 2024 Dec 4;15:10483. doi: 10.1038/s41467-024-54830-w (PMC11618350; doi:10.1038/s41467-024-54830-w)
Supplement: Supplementary file 6 — Reporting Summary [file 41467_2024_54830_MOESM6_ESM.pdf]

nature portfolio

Corresponding author(s): Konstantinos Ampatzis
Caghan Kizil

Last updated by author(s): Sep 22, 2024

Reporting Summary

Nature Portfolio wishes to improve the reproducibility of the work that we publish. This form provides structure for consistency and transparency in reporting. For further information on Nature Portfolio policies, see our Editorial Policies and the Editorial Policy Checklist.

Statistics

For all statistical analyses, confirm that the following items are present in the figure legend, table legend, main text, or Methods section.

n/a	Confirmed	
☐	☒	The exact sample size (*n*) for each experimental group/condition, given as a discrete number and unit of measurement
☐	☒	A statement on whether measurements were taken from distinct samples or whether the same sample was measured repeatedly
☐	☒	The statistical test(s) used AND whether they are one- or two-sided<br>*Only common tests should be described solely by name; describe more complex techniques in the Methods section.*
☒	☐	A description of all covariates tested
☒	☐	A description of any assumptions or corrections, such as tests of normality and adjustment for multiple comparisons
☐	☒	A full description of the statistical parameters including central tendency (e.g. means) or other basic estimates (e.g. regression coefficient) AND variation (e.g. standard deviation) or associated estimates of uncertainty (e.g. confidence intervals)
☐	☒	For null hypothesis testing, the test statistic (e.g. *F*, *t*, *r*) with confidence intervals, effect sizes, degrees of freedom and *P* value noted<br>*Give P values as exact values whenever suitable.*
☒	☐	For Bayesian analysis, information on the choice of priors and Markov chain Monte Carlo settings
☒	☐	For hierarchical and complex designs, identification of the appropriate level for tests and full reporting of outcomes
☒	☐	Estimates of effect sizes (e.g. Cohen's *d*, Pearson's *r*), indicating how they were calculated

Our web collection on statistics for biologists contains articles on many of the points above.

Software and code

Policy information about availability of computer code

Data collection	All electrophysiology data were collected using pCLAMP 11 software suite (Clampex; Molecular Devices). All confocal images were acquired using the ZEN software (ZEISS).
Data analysis	Electrophysiology data analyzed using the pCLAMP 11 software suite (Clampfit v11.0; Molecular Devices) and AxoGraph (version X 1.5.4; AxoGraph Scientific, Sydney, Australia; RRID: SCR_014284).<br>Confocal microscopy image acquisition with ZEN (version 3.4/blue edition)<br>Anatomy data were analyzed using ImageJ/Fiji software (version 2.1.0/1.53c; https://imagej.nih.gov/ij/) and Origin 8 (OriginLab, Northampton, MA, USA).<br>Statistical analysis performed using Prism 9.0 (GraphPad Software Inc).<br>All images and figures processed using Photoshop (version 21.0.0) and Illustrator (version 24.1; Adobe Systems Inc., San Jose, CA, USA)<br>The raw single-cell sequencing underwent analysis using the default settings of the<br>473 Cell Ranger Single Cell Software Suite (10X Genomics, version 6.1.2).We normalized and scaled the data and used FindNeighbours<br>494 with 30 dims. Clustering was performed using resolution 1.5. We identified four subclusters.<br>495 For the detection of average and percentage expression of selected genes, we used<br>496 AverageExpression and PrctCellExpringGene functions, respectively.

For manuscripts utilizing custom algorithms or software that are central to the research but not yet described in published literature, software must be made available to editors and reviewers. We strongly encourage code deposition in a community repository (e.g. GitHub). See the Nature Portfolio guidelines for submitting code & software for further information.

Data

Policy information about availability of data

All manuscripts must include a data availability statement. This statement should provide the following information, where applicable:

- Accession codes, unique identifiers, or web links for publicly available datasets
- A description of any restrictions on data availability
- For clinical datasets or third party data, please ensure that the statement adheres to our policy

Histology and electrophysiology data used in this study analyses are available in Supplementary table 1. No custom or open-source code was used for data collection and analyses. For single clel dataset, Seurat software and relevant R packages as detailed above were used. The single-cell sequencing dataset generated in this study has been deposited in the NCBI's GEO database under accession code GSE261619.

Research involving human participants, their data, or biological material

Policy information about studies with human participants or human data. See also policy information about sex, gender (identity/presentation), and sexual orientation and race, ethnicity and racism.

Reporting on sex and gender	Animals (zebrafish) of both sexes were used in this study.
Reporting on race, ethnicity, or other socially relevant groupings	N/A
Population characteristics	N/A
Recruitment	N/A
Ethics oversight	N/A

Note that full information on the approval of the study protocol must also be provided in the manuscript.

Field-specific reporting

Please select the one below that is the best fit for your research. If you are not sure, read the appropriate sections before making your selection.

☒ Life sciences ☐ Behavioural & social sciences ☐ Ecological, evolutionary & environmental sciences

For a reference copy of the document with all sections, see nature.com/documents/nr-reporting-summary-flat.pdf

Life sciences study design

All studies must disclose on these points even when the disclosure is negative.

Sample size	No statistical method was used to predetermine sample size. Sample sizes were chosen based on published studies and reported power analyses in the field, and are comparable to those in our previous publications (Chang, W., et al. (2021). Nature Communications 12, 4857; Chang, W., et al. (2020). Proc. Natl. Acad. Sci. U.S.A. 117, 17330–17337; Pedroni, A., and Ampatzis, K. (2019). iScience 19, 1189–1201; Bertuzzi, M., Chang, W., and Ampatzis, K. (2018). Proc. Natl. Acad. Sci. U.S.A. 115, E9926–E9933; Bertuzzi, M., and Ampatzis, K. (2018). Spinal cholinergic interneurons differentially control motoneuron excitability and alter the locomotor network operational range. Sci Rep 8, 1988.). Sample sizes are typically determined by the minimum number of animals needed for statistical significance, reducing unnecessary animal use. For single cell dataset, SCOPIT and scPower was used to determine the effect size and power.
Data exclusions	Zebrafish showing incorrect locomotor behavior and posture, and signs of significant distress were excluded from experiments.
Replication	The number of independently replicated experiments is described in the figure legends and text. All replication attempts were successful between different researchers.
Randomization	Zebrafish were randomly allocated into different experimental groups. No specific randomization method was used. Age/size-matched animals were used as controls in all experiments.
Blinding	All manual counts of HuC/D, NeuN, ChAT, Gad1, TH, GABA, vGluT1, vGluT2a cells were performed in a manner where the experimenters were blind to the conditions. In all quantifications, multiple investigators participated independently to ensure the reproducibility of the data. The experimenters were not blind to the experimental groups in the case of all electrophysiological recordings as the investigators (n=2) performed visually guided recordings of selective Eval3:GFP neurons located in the heart, using whole mound preparations to visualize and identify the SAP area. Thus, blinding was not applicable. Moreover, the functional studies using the ex-vivo preparations (intact and reduced) did not include blind approaches because the experiments were performed in a sequential manner (temporal investigations and pharmacological administration).

Reporting for specific materials, systems and methods

We require information from authors about some types of materials, experimental systems and methods used in many studies. Here, indicate whether each material, system or method listed is relevant to your study. If you are not sure if a list item applies to your research, read the appropriate section before selecting a response.

Materials & experimental systems

n/a	Involved in the study
☐	☒ Antibodies
☒	☐ Eukaryotic cell lines
☒	☐ Palaeontology and archaeology
☐	☒ Animals and other organisms
☒	☐ Clinical data
☒	☐ Dual use research of concern
☒	☐ Plants

Methods

n/a	Involved in the study
☒	☐ ChIP-seq
☐	☒ Flow cytometry
☒	☐ MRI-based neuroimaging

Antibodies

Antibodies used

All antibodies (primary and secondary) used in this study, along with details about the species raised, dilution used, and RRID numbers, are provided in great detail in Supplementary Table 2.

Validation

All primary antibodies have been validated extensively in numerous previous publications for zebrafish and other species.

CChAT (goat): It is a widely used marker for detecting cholinergic neurons in numerous species, with over 270 citations. The antibody was used extensively in zebrafish from our lab and others (Chang, W., et al., 2021. Nature Communications 12, 4857; Pedroni and Ampatzis, 2019. iScience; Berg et al., 2018. Brain Struct Funct; Bertuzzi and Ampatzis, 2018. Sci Rep; DeMarko et al., 2019. Journal of Comparative Neurology; DeOliveira-Mello, 2019. Brain Res.) with 53 zebrafish references (http://zfin.org/ZDB-ATB-081017-3#summary).

GABA: widely used marker for detection of GABAergic neurons with 147 references (https://scicrunch.org/resources/Any/search?q=AB_477652&l=AB_477652). In zebrafish, the field is extensively used to detect GABAergic neurons in both the brain and spinal cord from our lab (Pedroni and Ampatzis, 2019. iScience; Berg et al., 2018. Brain Struct Funct) and others with 44 references (http://zfin.org/ZDB-ATB-081106-2#summary).

GFP (chicken): It is a widely used marker for detecting GFP in several models, including the zebrafish with more than 490 citations including zebrafish (Hall et al., 2009, Journal of Leukocyte Biology; Lee at al., 2013, Development; Groteck et al., 2013, Development).

HuC/D (mouse): A widely used marker for detecting eval3&4 neuronal proteins and therefore an accurate neuronal marker. The antibody has 464 citations in zebrafish (http://zfin.org/ZDB-ATB-081003-2#summary) and another 300 citations in other animals.

HuC/D (rabbit): A zebrafish specific antibody against the neuronal proteins Elav3+4 (https://www.genetex.com/Product/Detail/Elavl3-4-antibody/GTX128365). As such, the manufacturer validated the antibody. The antibody used before in zebrafish spinal cord (Pedroni and Ampatzis, 2019. iScience; Berg et al., 2018. Brain Struct Funct; Li et al., 2019. Stem cells research & therapy).

NeuN (Rabbit): A widely used monoclonal antibody for the detection of the neuronal nuclei marker Fox-3 (RBFOX3), which is a nuclear protein expressed in post-mitotic neurons of the central and peripheral nervous systems. The NeuN antibody has more than 180 citations, and we used it before Chang et al., 2021 (Nature Communications 12:4857).

Zn-12 (Mouse): A monoclonal (mouse) neuronal cell surface marker against bot sulfated and non-sulfated HNK-1 carbohydrate epitopes. The Zn-12 antibody has been used in 129 publications in zebrafish model system (https://zfin.org/ZDB-ATB-081002-12#summary).

TH (Mouse): Monoclonal antibody against tyrosine hydroxylase. Is a widely used antibody in several previous studies in various model systems. In the zebrafish has been used in 114 publications (https://zfin.org/ZDB-ATB-090209-1#anatomical-labeling).

5-HT (Rabbit): Polyclonal antibody against serotonin used before to describe the serotonergic neurons in the zebrafish spinal cord (Pedroni and Ampatzis, 2019). The antibody in zebrafish has 69 citations (https://zfin.org/ZDB-ATB-081017-7#summary).

A2B-Ars (Mouse): Antibody against alpha2-adrenergic receptors, a member of the G protein-coupled receptor family. The manufacturer validated the specificity of the antibody using western blot analyses. A new antibody has been used in a small number of studies (#15); however, the specificity against the alpha2B adrenergic receptor is well described.

Gad1b (Rabbit): Zebrafish-specific polyclonal antibody against Gad1b that corresponds to mammalian GAD67. The manufacturer validated the specificity of the antibody using western blot analyses and immunostainings in zebrafish (https://www.genetex.com/PDF/Download?catno=GTX124355).

Animals and other research organisms

Policy information about studies involving animals; ARRIVE guidelines recommended for reporting animal research, and Sex and Gender in Research

Laboratory animals	Young adult zebrafish (8–12 weeks old with total length > 1.8-2.0 cm post fertilization) of both sexes were used for anatomical,and electrophysiological, and heart functionality studies. Older animals were used for sequencing experiments<br>Wild type zebrafish: AB/Tübingen, RRID: ZIRC_ZL1.<br>Transgenic zebrafish: elavl3:eGFP, ZDB-FISH-220124-10; vGluT2a:DsRed, RRID:ZDB-FISH-150901-14045; Gad1b:GFP, RRID:ZDB-FISH-150901-12650; nbt:DsRed, RRID:ZDB-FISH-150901-3075<br>More details regarding the age, size, sex and number used in this study are provided in the manuscript text and Methods section.
Wild animals	This study did not involve wild animals.
Reporting on sex	Adult wild-type zebrafish (Danio rerio) a of both sexes were used in this study. Although sexual dimorphisms exist in the adult zebrafish morphology and behavior, this animal model lacks a defined sex determination mechanism (e.g., SRY) or discernible sex chromosomes and sex-related genes. The present study focuses on characterizing the intracardiac nervous system and their involvement in heart functionality. Examining animal factors, such as sex, is beyond the study's scope as the goal is to provide insight into the principal characteristics of the intra-cardiac neurons.
Field-collected samples	This study did not involve samples collected from the field.
Ethics oversight	The Regional Animal Research Ethical Committee Stockholm (for Karolinska Institutet) approved all experimental protocols, Stockholm (Ethical permit no. 13913-2022), and were implemented under EU guidelines for the care and use of laboratory animals (2010/63/EU) and directed according to the ARRIVE guidelines.

Note that full information on the approval of the study protocol must also be provided in the manuscript.

Flow Cytometry

Plots

Confirm that:

☒ The axis labels state the marker and fluorochrome used (e.g. CD4-FITC).

☒ The axis scales are clearly visible. Include numbers along axes only for bottom left plot of group (a 'group' is an analysis of identical markers).

☒ All plots are contour plots with outliers or pseudocolor plots.

☒ A numerical value for number of cells or percentage (with statistics) is provided.

Methodology

Sample preparation	Following dissociation, cells underwent filtration using a 40 µM cell strainer into a solution comprising 10 mL of 2% BSA in PBS. Centrifugation at 300 g for 10 minutes was followed by resuspension in a cell suspension buffer containing 2% BSA in PBS. This cell suspension was used to sort viable GFP-positive and dsRED-positive cells as singlets using an appropriate gating strategy (Supplementary Figure 5, Supplementary Data 1) together with viability indicator dyes Sytox Blue (Invitrogen, Cat No. S34857) and Dycle Ruby (Invitrogen, Cat. No. V10309) by FACS. The single-cell suspension was promptly loaded onto the 10X Chromium system (Zheng et al., 2016). The 10X libraries were sequenced via Illumina NovaSeq 6000 (Bhattarai et al., 2016; Cosacak et al., 2019; Cosacak et al., 2020; Bhattarai et al., 2020. In total, 9,997 cells were sequenced.
Instrument	Sony MA900-FP
Software	Sony MA900-FP
Cell population abundance	In total, 9,997 cells were sequenced.
Gating strategy	Gating strategy is summarized in Supplementary Figure 5, Supplementary Data 1. Cells were sorted together with viability indicator dyes Sytox Blue (Invitrogen, Cat No. S34857) and Dycle Ruby (Invitrogen, Cat. No. V10309) by FACS.

☒ Tick this box to confirm that a figure exemplifying the gating strategy is provided in the Supplementary Information.
